# Crystal structure of aqua­chlorido­(nitrato-κ^2^ *O*,*O*′)[1-(pyridin-2-yl-κ*N*)-2-(pyridin-2-yl­methyl­idene-κ*N*)hydrazine-κ*N* ^2^]manganase(II)

**DOI:** 10.1107/S2056989018003493

**Published:** 2018-03-06

**Authors:** Mamour Sarr, Mayoro Diop, Elhadj Ibrahima Thiam, Aliou Hamady Barry, Mohamed Gaye, Pascal Retailleau

**Affiliations:** a Département de Chimie, Faculté des Sciences et Techniques, Université Cheikh Anta Diop, Dakar, Senegal; b Département de Chimie, Faculté des Sciences, Université de Nouakchott, Nouakchott, Mauritania; c Centre de Recherche e Gif, Institut de Chimie des Substances Naturelles, CNRS-UPR2301, 1 Avenue la Terasse, 91198 Gif sur Yvette, France

**Keywords:** crystal structure, manganese, Schiff base

## Abstract

The search for novel manganese(II) compounds having inter­esting magnetic properties, using 1-(pyridin-2-yl)-2-(pyridin-2-yl­methyl­idene)hydrazine (H*L*) as a tridendate ligand, led to the preparation of the title mononuclear material, [MnCl(NO_3_)(C_11_H_10_N_4_)(H_2_O)], and the determination of its structure by XRD. The asymmetric unit comprises a discrete mol­ecule in which the cation Mn^II^ is hepta­coordinated. The environment around the cation is an almost perfect penta­gonal bipyramid. The base is defined by the two N atoms of the pyridine rings, the N atom of the imino function of the ligand and the two O atoms of the chelating bidentate nitrate ligand. The apical positions are occupied by a Cl atom and a water mol­ecule. In the crystal, there are numerous hydrogen bonds of the types O*w*—H⋯ONO_2_ and C—H⋯ONO_2_, which generate layers parallel to the *bc* plane in which the ligands in the axial positions point into the inter­layer space. These axial ligands give rise to hydrogen bonds of the types O*w*—H⋯ONO_2_, O*w*—H⋯Cl, N—H⋯Cl and C—H⋯Cl, leading to a three-dimensional framework. The chain bridging the two pyridine rings is disordered over two sets of sites in a 0.53 (2):0.47 (2) ratio.

## Abstract 2

The asymmetric unit comprises a discrete mol­ecule in which the cation Mn^II^ is hepta­coordinated. The environment around the cation is an almost perfect penta­gonal bipyramid. In the crystal, extensive hydrogen bonding leads to a three-dimensional framework.

## Chemical context   

Although very much studied, the coordination chemistry of manganese remains very inter­esting as this metal can have several degrees of oxidation and its complexes can display different coordination numbers and geometries that are not always easily predicted (Chiswell *et al.*, 1987 ▸; Baldeau *et al.*, 2004 ▸; Mikuriya *et al.*, 1997 ▸). Although the coordination numbers four and six are the most common in the coordin­ation chemistry of manganese, the coordination numbers five, seven and eight are also observed (Louloudi *et al.*, 1999 ▸). As a result of the multiple degrees of oxidation of this metal, inter­est in the coordination chemistry of manganese complexes is considerable. The involvement of manganese in various important biological processes such as oxidation of water by photosynthetic enzymes (Whittaker & Whittaker, 1997 ▸), hydrogen peroxide disproportionation by catalase (Meier *et al.*, 1996 ▸), superoxide dismutase (SOD) (Schwartz *et al.*, 2000 ▸), ribonucleotide reductase and lipoxygenase (Baffert *et al.*, 2003 ▸) increases the inter­est of scientists in this metal. These examples from nature inspire chemists to search for bio-mimetic catalysts of these metalloenzymes that are highly selective and cause little damage to the environment (Krishnan & Vancheesan, 1999 ▸). Manganese complexes are also used as catalysts in many processes such as epoxidation of alkene (Castaman *et al.*, 2009 ▸), oxidation (Wegermann *et al.*, 2014 ▸) and hydrogenation of ketones (Bruneau-Voisine *et al.*, 2017 ▸). The involvement of the metal center in these processes depends as much on its degree of oxidation as on its coordination number in the complex. The synthetic procedures adopted are essential for yielding complexes with specific properties. In this context, for the synthesis of the hepta­coordinated Mn^II^ title complex, we use a one-pot synthesis method, which is an efficient approach to prepare a large variety of coordination compounds (Oyaizu *et al.*, 2000 ▸). Manganese dichloride tetra­hydrate is mixed with the synthesized organic ligand (H*L*), which provides three soft nitro­gen-binding sites in the presence of nitrate anions that can act with hard oxygen-binding sites to yield a mononuclear hepta­coordinated manganese(II) complex.
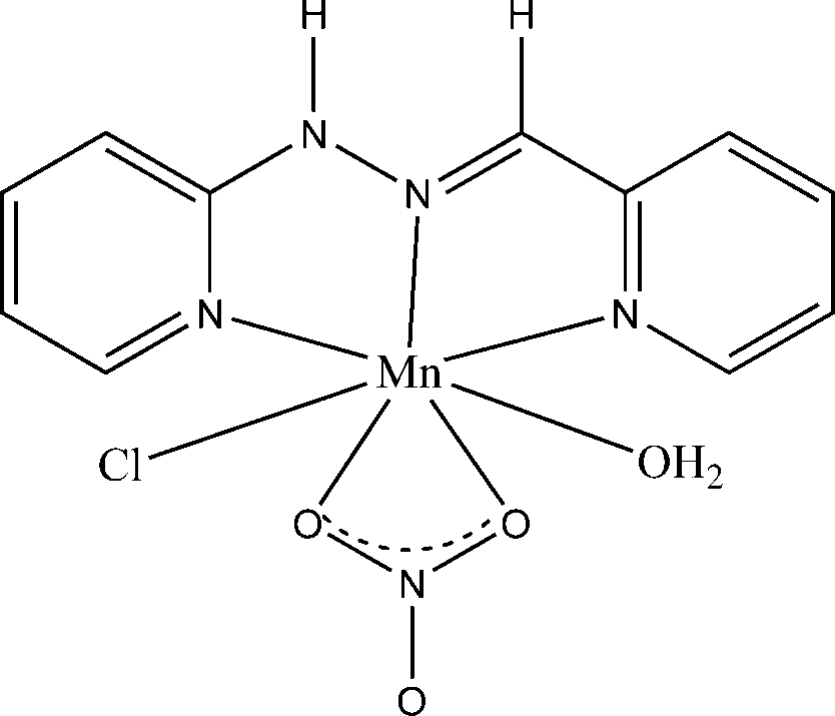


## Structural commentary   

**Figure 1:**
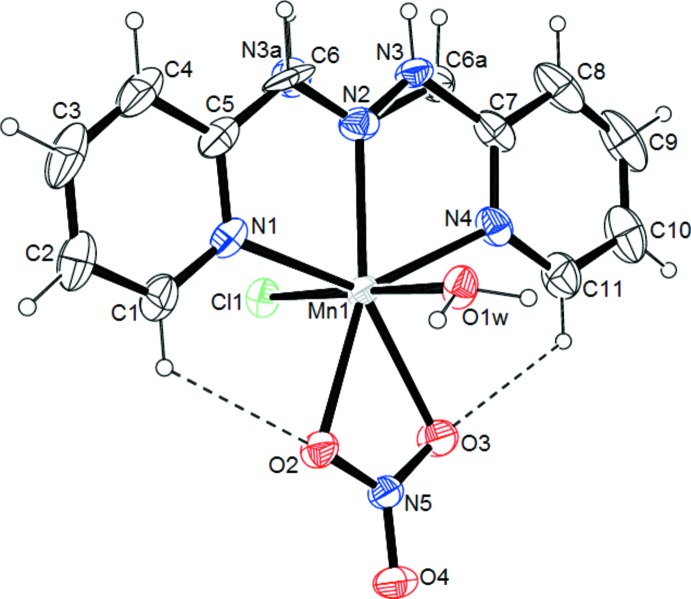
An *ORTEP* view of the title compound, showing the atom-numbering scheme and intra­molecular hydrogen bonds as dashed lines. Displacement ellipsoids are plotted at the 50% probability level.

The structure of the title complex is shown in Fig. 1 ▸. The asymmetric unit comprises a discrete mol­ecule in which the cation Mn^II^ is hepta­coordinated. The coordination polyhedron of the Mn^II^ center is best described as a distorted penta­gonal bipyramid with an MnN_3_O_3_Cl chromophore. The basal plane is occupied by two nitro­gen atoms from the pyridine rings, one nitro­gen atom from the imino function and two oxygen atoms from the chelating bidentate nitrate group. The metal-bound ligand nitro­gen atoms exhibit angles of 69.85 (7)° (N1—Mn1—N2) and 69.62 (7)° (N2—Mn1—N4) which are slightly different from the ideal angle for a regular penta­gon (72°). The sum of the equatorial angles around Mn^II^ is 359.72°. The angle formed by the atoms in axial positions around Mn^II^ [Cl1—Mn1—O1*W* = 179.07 (4)°] is very close to the ideal value of 180°. The Mn—O/N bond lengths (Table 1 ▸) are longer than those observed in the hepta­dentate manganese complex [Mn(*L*)(NO_3_)_2_] [*L* is 2,6-bis­(1-butyl-1*H*-benzo[*d*]imidazol-2-yl)pyridine; Kose & McKee *et al.*, 2014 ▸]. The apical bond Mn1—Cl1 [2.4999 (6) Å] is the longest and is comparable to those found for the complex [Mn(*L*)(Cl)_2_]·MeOH [Mn1—Cl1 = 2.4849 (7), Mn1—Cl2 = 2.5465 (7) Å] {*L* is 2,6-bis­[(2-hy­droxy­phenyl­imino)­meth­yl]pyridine; Kose *et al.*, 2015 ▸}. The second axial bond is the shortest distance in the structure [Mn1—O1*W* = 2.2239 (14) Å]. The two pyridine rings are connected by a disordered chain C—CH=N—NH—C in which the bond lengths are slightly different from those observed in similar complexes; this may be related to the observed disorder. Two intra­molecular hydrogen bonds, C1—H1⋯O2 and C11—H11⋯O3, are also observed in the structure (Table 2 ▸, Fig. 1 ▸).

## Supra­molecular features   

**Figure 2:**
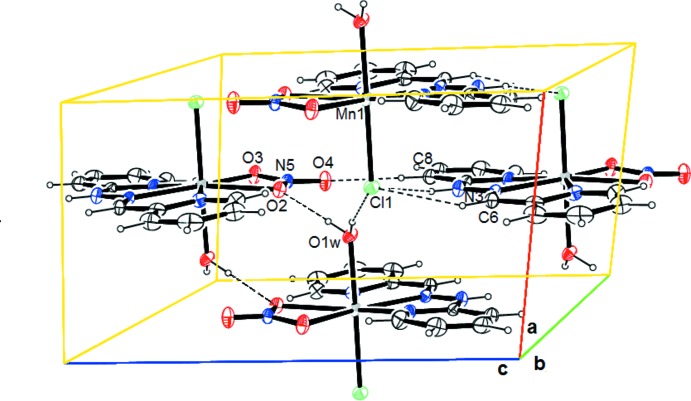
Representation of the title compound, showing the inter­molecular hydrogen bonds as dashed lines.

In the crystal, the complex mol­ecules are linked by hydrogen bonds, giving rise to a three-dimensional network (Fig. 2 ▸, Table 2 ▸). The structure is built up from penta­gonal bipyramids around the Mn^II^ atom, which are assembled in layers parallel to the *bc* plane. These layers are inter­connected by hydrogen bonds. The coordinating axial water mol­ecule points into the inter­layer space and act as a hydrogen-bond donor towards oxygen atom O2-NO_2_ and chlorine atom Cl1 (Fig. 2 ▸) *via* the hydrogen bonds O1*W*—H1*WB*⋯O2^ii^ and O1*W*—H1*WA*⋯Cl1^i^, [symmetry codes: (i) *x* + 1, *y*, *z*; (ii) −*x* + 2, −*y* + 1, −*z* + 2]. The axial Cl1 atom points also in the inter­layer space and acts as a hydrogen-bond acceptor toward N3—H3*N*⋯Cl1^iii^ and C6—H6⋯Cl1^iii^ [symmetry code: (iii) −*x* + 1, −*y* + 1, −*z* + 1]. The combined hydrogen bonds link the layers into a three-dimensional framework. Within a layer, the mol­ecules are inter­connected by hydrogen bonds of the type C—H⋯ONO_2_ [C8—H8⋯O4^iv^—NO_2_; symmetry code: (iv) *x*, *y*, *z* − 1].

## Database survey   

The ligand 1-(2-pyridyl)-2-(pyridin-2-yl­methyl­idene)hydrazine has been widely used in coordination chemistry. The current release of the CSD (Version 5.39, last update Nov 2017; Groom *et al.*, 2016 ▸) affords 22 hits. Seven examples of complexes with the above ligand with *f*-block metal ions appear in the literature (Baraniak *et al.*, 1976 ▸; Ndiaye-Gueye, Dieng, Thiam, Sow *et al.*, 2017 ▸; Ndiaye-Gueye, Dieng, Thiam, Lo *et al.*, 2017 ▸; Ndiaye-Gueye, Dieng, Lo *et al.*, 2017 ▸; Gueye *et al.*, 2017 ▸). The other entries are for complexes with *p*- and *d*-block metal ions. Structures are available for Ca^II^ (Vantomme *et al.*, 2014 ▸ ▸), Cu^II^ (Mesa *et al.*, 1988 ▸, 1989 ▸; Rojo *et al.*, 1988 ▸; Ainscough *et al.*, 1996 ▸; Chowdhury *et al.*, 2009 ▸; Mukherjee *et al.*, 2010 ▸; Chang *et al.*, 2011 ▸), Co^II^ (Gerloch *et al.*, 1966 ▸), Ni^II^ (Chiumia *et al.*, 1999 ▸) and Zn^II^ (Dumitru *et al.*, 2005 ▸; Vantomme *et al.*, 2014 ▸ ▸). In all cases, the ligand behaves as a tridentate ligand acting through the soft nitro­gen donor atoms from the two pyridine rings and the imino function. The hard protonated nitro­gen atom remains uncoordinated in all complexes.

## Synthesis and crystallization   

A mixture of 2-hydrazino­pyridine (1 mmol) and 2-pyridine­carbaldehyde (1 mmol) in ethanol (10 mL) was stirred under reflux for 30 min. A mixture of ammonium nitrate (3 mmol) and MnCl_2_·4H_2_O (1 mmol) in ethanol (10 mL) was added to the solution. The mixture was stirred for 30 min and the resulting yellow solution was filtered and the filtrate was kept at 298 K. A yellow powder appeared after one day and was collected by filtration, yield 65%. Analysis calculated for [C_11_H_12_ClMnN_5_O_4_] C, 32.41; H, 3.26; N, 22.67. Found: C, 32.37; H, 3.19; N, 22.60%. μ_eff_(μ_B_): 5.98 Λ_M_ (S cm^2^ mol^−1^): 14. IR (cm^−1^): 3233, 1609, 1560, 1521, 1465, 1422, 1289, 1148, 776, 673.

## Refinement   

Crystal data, data collection and structure refinement details are summarized in Table 3 ▸. All H atoms (=CH, NH and OH_2_ groups) were optimized geometrically (C—H = 0.93, N—H = 0.86 and O—H = 0.87–0.91 Å) and refined as riding with *U*_iso_(H) = 1.2*U*_eq_(C) or 1.5*U*_eq_(O). The chain bridging the two pyridine rings is disordered. This disorder may be explained by the fact that the sequence of atoms C(Py)—CH=N—NH—C(py) overlaps with the sequence C(py)—NH—N=CH—C(py), meaning two orientations of the ligand. In such a case, for the refinement it was assumed that the C atom of the CH group from one chain and the NH atoms from the second chain occupy the same position. The same relates inversely. The occupancy factor refined to 0.53 (2):0.47 (2).

## Supplementary Material

Crystal structure: contains datablock(s) I. DOI: 10.1107/S2056989018003493/eb2005sup1.cif

Structure factors: contains datablock(s) I. DOI: 10.1107/S2056989018003493/eb2005Isup2.hkl

CCDC reference: 1826459

Additional supporting information:  crystallographic information; 3D view; checkCIF report

## Figures and Tables

**Table 1:** Selected bond lengths (Å)

Mn1—O1*W*	2.2239 (14)	N2—N3*A*	1.288 (11)
Mn1—N2	2.2750 (16)	N2—C6	1.325 (13)
Mn1—N4	2.3292 (16)	N2—C6*A*	1.465 (10)
Mn1—N1	2.3300 (16)	N3—C7	1.566 (9)
Mn1—O2	2.3372 (14)	C5—C6	1.398 (15)
Mn1—O3	2.3635 (15)	C5—N3*A*	1.450 (11)
Mn1—Cl1	2.4999 (6)	C7—C6*A*	1.180 (11)
N2—N3	1.217 (7)		

**Table 2:** Hydrogen-bond geometry (Å, °)

*D*—H⋯*A*	*D*—H	H⋯*A*	*D*⋯*A*	*D*—H⋯*A*
O1*W*—H1*WA*⋯Cl1^i^	0.91	2.23	3.1225 (15)	170
O1*W*—H1*WB*⋯O2^ii^	0.87	1.92	2.7969 (19)	177
O1*W*—H1*WB*⋯N5^ii^	0.87	2.68	3.506 (2)	157
N3—H3*N*⋯Cl1^iii^	0.86	2.71	3.501 (7)	153
C1—H1⋯O2	0.93	2.53	3.140 (3)	124
C6—H6⋯Cl1^iii^	0.93	2.66	3.489 (11)	149
C8—H8⋯O4^iv^	0.93	2.54	3.290 (3)	138
C10—H10⋯Cl1^v^	0.93	2.83	3.669 (3)	152
C11—H11⋯O3	0.93	2.44	3.062 (3)	125

Symmetry codes: (i) 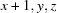; (ii) 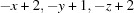; (iii) 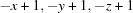; (iv) 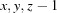; (v) 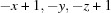.

**Table 3:** Experimental details

Crystal data	
Chemical formula	[MnCl(NO_3_)(C_11_H_10_N_4_)(H_2_O)]
*M*_r_	368.65
Crystal system, space group	Triclinic, *P* 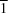
Temperature (K)	293
*a*, *b*, *c* (Å)	6.9698 (1), 10.6055 (2), 10.8476 (2)
α, β, γ (°)	98.784 (2), 97.636 (2), 108.308 (2)
*V* (Å^3^)	738.21 (2)
*Z*	2
Radiation type	Mo *K*α
μ (mm^−1^)	1.10
Crystal size (mm)	0.09 × 0.08 × 0.06

Data collection	
Diffractometer	Bruker KappaCCD
Absorption correction	–
No. of measured, independent and observed [*I* > 2σ(*I*)] reflections	22026, 3571, 2905
*R*_int_	0.038

Refinement	
*R*[*F* ^2^ > 2σ(*F* ^2^)], *wR*(*F* ^2^), *S*	0.034, 0.091, 1.06
No. of reflections	3571
No. of parameters	209
H-atom treatment	H-atom parameters constrained
Δρ_max_, Δρ_min_ (e Å^−3^)	0.31, −0.46

Computer programs: *APEX3* and *SAINT* (Bruker, 2016 ▸), *SHELXT* (Sheldrick, 2015*a* ▸), *SHELXL2014* (Sheldrick, 2015*b* ▸) and *ORTEP-3 for Windows* (Farrugia, 2012 ▸).

## supplementary crystallographic information

### Crystal data

**Table:**

[MnCl(NO_3_)(C_11_H_10_N_4_)(H_2_O)]	*Z* = 2
*M**_r_* = 368.65	*F*(000) = 374
Triclinic, *P*1	*D*_x_ = 1.658 Mg m^−^^3^
*a* = 6.9698 (1) Å	Mo *K*α radiation, λ = 0.71073 Å
*b* = 10.6055 (2) Å	Cell parameters from 9920 reflections
*c* = 10.8476 (2) Å	θ = 2.4–28.6°
α = 98.784 (2)°	µ = 1.10 mm^−^^1^
β = 97.636 (2)°	*T* = 293 K
γ = 108.308 (2)°	Prismatic, yellow
*V* = 738.21 (2) Å^3^	0.09 × 0.08 × 0.06 mm

### Data collection

**Table:**

Bruker KappaCCD diffractometer	2905 reflections with *I* > 2σ(*I*)
Radiation source: fine-focus sealed tube	*R*_int_ = 0.038
Detector resolution: 9 pixels mm^-1^	θ_max_ = 29.1°, θ_min_ = 3.6°
CCD scans	*h* = −9→9
22026 measured reflections	*k* = −14→14
3571 independent reflections	*l* = −14→14

### Refinement

**Table:**

Refinement on *F*^2^	Primary atom site location: structure-invariant direct methods
Least-squares matrix: full	Secondary atom site location: difference Fourier map
*R*[*F*^2^ > 2σ(*F*^2^)] = 0.034	Hydrogen site location: mixed
*wR*(*F*^2^) = 0.091	H-atom parameters constrained
*S* = 1.06	*w* = 1/[σ^2^(*F*_o_^2^) + (0.0431*P*)^2^ + 0.1976*P*] where *P* = (*F*_o_^2^ + 2*F*_c_^2^)/3
3571 reflections	(Δ/σ)_max_ = 0.001
209 parameters	Δρ_max_ = 0.31 e Å^−^^3^
0 restraints	Δρ_min_ = −0.46 e Å^−^^3^

### Special details

**Table:**

Geometry. All esds (except the esd in the dihedral angle between two l.s. planes) are estimated using the full covariance matrix. The cell esds are taken into account individually in the estimation of esds in distances, angles and torsion angles; correlations between esds in cell parameters are only used when they are defined by crystal symmetry. An approximate (isotropic) treatment of cell esds is used for estimating esds involving l.s. planes.

### Fractional atomic coordinates and isotropic or equivalent isotropic displacement parameters (Å^2^)

**Table:**

	*x*	*y*	*z*	*U*_iso_*/*U*_eq_	Occ. (<1)
Mn1	0.71914 (4)	0.39182 (3)	0.74637 (2)	0.04153 (11)	
Cl1	0.33417 (8)	0.30415 (5)	0.69679 (5)	0.05511 (14)	
O1W	1.0615 (2)	0.46771 (14)	0.79278 (13)	0.0541 (3)	
H1WA	1.140892	0.416765	0.774402	0.081*	
H1WB	1.118139	0.516748	0.869134	0.081*	
O2	0.7483 (2)	0.38248 (13)	0.96166 (13)	0.0529 (3)	
O3	0.7175 (3)	0.19991 (15)	0.83256 (13)	0.0631 (4)	
O4	0.7546 (3)	0.20477 (18)	1.03568 (15)	0.0728 (5)	
N1	0.7321 (3)	0.61349 (16)	0.81806 (18)	0.0528 (4)	
N2	0.7448 (2)	0.50621 (19)	0.58471 (17)	0.0519 (4)	
N3	0.7437 (8)	0.4569 (8)	0.4755 (5)	0.0484 (13)	0.53 (2)
H3N	0.742706	0.499635	0.414304	0.058*	0.53 (2)
N4	0.7511 (3)	0.25742 (19)	0.56463 (15)	0.0550 (4)	
N5	0.7400 (3)	0.25956 (16)	0.94508 (15)	0.0474 (4)	
C1	0.7239 (4)	0.6682 (2)	0.9358 (2)	0.0651 (6)	
H1	0.706417	0.613021	0.995278	0.078*	
C2	0.7400 (4)	0.8019 (2)	0.9744 (3)	0.0810 (8)	
H2	0.735126	0.835887	1.057767	0.097*	
C3	0.7630 (5)	0.8828 (3)	0.8875 (4)	0.0962 (11)	
H3	0.773464	0.973219	0.910758	0.115*	
C4	0.7707 (4)	0.8300 (3)	0.7648 (4)	0.0840 (9)	
H4	0.787019	0.884113	0.704332	0.101*	
C5	0.7536 (3)	0.6938 (2)	0.7329 (3)	0.0593 (6)	
C6	0.757 (2)	0.6350 (13)	0.6090 (13)	0.072 (6)	0.53 (2)
H6	0.766705	0.684993	0.545312	0.087*	0.53 (2)
C7	0.7444 (3)	0.3077 (3)	0.45798 (19)	0.0605 (6)	
C8	0.7378 (4)	0.2313 (4)	0.3399 (2)	0.0898 (10)	
H8	0.732275	0.267981	0.267437	0.108*	
C9	0.7394 (5)	0.1032 (4)	0.3320 (3)	0.1050 (13)	
H9	0.732556	0.050255	0.253735	0.126*	
C10	0.7511 (5)	0.0521 (3)	0.4395 (3)	0.0934 (10)	
H10	0.754750	−0.035280	0.435722	0.112*	
C11	0.7575 (4)	0.1323 (3)	0.5545 (2)	0.0744 (7)	
H11	0.766778	0.097228	0.627718	0.089*	
C6A	0.7462 (14)	0.4203 (12)	0.4658 (9)	0.051 (2)*	0.47 (2)
H6A	0.748686	0.456233	0.392738	0.061*	0.47 (2)
N3A	0.755 (2)	0.6312 (11)	0.6045 (10)	0.057 (4)*	0.47 (2)
H3NA	0.761514	0.675558	0.544192	0.069*	0.47 (2)

### Atomic displacement parameters (Å^2^)

**Table:**

	*U*^11^	*U*^22^	*U*^33^	*U*^12^	*U*^13^	*U*^23^
Mn1	0.05255 (18)	0.03866 (16)	0.03561 (16)	0.02057 (12)	0.00415 (12)	0.00731 (11)
Cl1	0.0516 (3)	0.0560 (3)	0.0593 (3)	0.0232 (2)	0.0087 (2)	0.0079 (2)
O1W	0.0570 (8)	0.0574 (8)	0.0463 (7)	0.0285 (7)	−0.0016 (6)	−0.0033 (6)
O2	0.0720 (9)	0.0401 (7)	0.0464 (7)	0.0235 (6)	0.0058 (7)	0.0038 (6)
O3	0.0995 (12)	0.0490 (8)	0.0421 (8)	0.0330 (8)	0.0078 (8)	0.0023 (6)
O4	0.1101 (13)	0.0758 (10)	0.0518 (9)	0.0480 (10)	0.0203 (9)	0.0308 (8)
N1	0.0537 (9)	0.0395 (8)	0.0670 (11)	0.0204 (7)	0.0041 (8)	0.0132 (8)
N2	0.0423 (8)	0.0652 (11)	0.0491 (10)	0.0171 (8)	0.0027 (7)	0.0224 (8)
N3	0.069 (3)	0.046 (3)	0.034 (2)	0.026 (2)	0.0046 (15)	0.014 (2)
N4	0.0540 (9)	0.0652 (11)	0.0392 (9)	0.0180 (8)	0.0063 (7)	−0.0017 (7)
N5	0.0609 (10)	0.0437 (8)	0.0407 (8)	0.0230 (7)	0.0073 (7)	0.0091 (7)
C1	0.0751 (15)	0.0483 (11)	0.0736 (15)	0.0301 (11)	0.0082 (12)	0.0037 (10)
C2	0.0791 (16)	0.0505 (13)	0.111 (2)	0.0316 (12)	0.0110 (15)	−0.0054 (14)
C3	0.0790 (18)	0.0406 (12)	0.170 (3)	0.0292 (12)	0.019 (2)	0.0101 (17)
C4	0.0739 (16)	0.0532 (13)	0.139 (3)	0.0303 (12)	0.0211 (17)	0.0425 (17)
C5	0.0462 (11)	0.0481 (11)	0.0895 (18)	0.0194 (9)	0.0080 (11)	0.0286 (11)
C6	0.067 (5)	0.071 (6)	0.095 (9)	0.028 (3)	0.003 (3)	0.067 (7)
C7	0.0391 (10)	0.0915 (18)	0.0366 (10)	0.0094 (10)	0.0041 (8)	0.0010 (10)
C8	0.0651 (15)	0.134 (3)	0.0410 (13)	0.0061 (17)	0.0118 (11)	−0.0108 (15)
C9	0.0724 (18)	0.132 (3)	0.0646 (19)	−0.0014 (18)	0.0206 (14)	−0.0446 (19)
C10	0.091 (2)	0.0782 (18)	0.091 (2)	0.0175 (15)	0.0285 (17)	−0.0271 (16)
C11	0.0906 (18)	0.0643 (14)	0.0637 (15)	0.0286 (13)	0.0194 (13)	−0.0085 (11)

### Geometric parameters (Å, º)

**Table:**

Mn1—O1W	2.2239 (14)	C1—C2	1.380 (3)
Mn1—N2	2.2750 (16)	C1—H1	0.9300
Mn1—N4	2.3292 (16)	C2—C3	1.361 (5)
Mn1—N1	2.3300 (16)	C2—H2	0.9300
Mn1—O2	2.3372 (14)	C3—C4	1.378 (5)
Mn1—O3	2.3635 (15)	C3—H3	0.9300
Mn1—Cl1	2.4999 (6)	C4—C5	1.396 (3)
O1W—H1WA	0.9067	C4—H4	0.9300
O1W—H1WB	0.8745	C5—C6	1.398 (15)
O2—N5	1.270 (2)	C5—N3A	1.450 (11)
O3—N5	1.251 (2)	C6—H6	0.9300
O4—N5	1.224 (2)	C7—C6A	1.180 (11)
N1—C1	1.337 (3)	C7—C8	1.394 (3)
N1—C5	1.341 (3)	C8—C9	1.352 (5)
N2—N3	1.217 (7)	C8—H8	0.9300
N2—N3A	1.288 (11)	C9—C10	1.362 (5)
N2—C6	1.325 (13)	C9—H9	0.9300
N2—C6A	1.465 (10)	C10—C11	1.386 (3)
N3—C7	1.566 (9)	C10—H10	0.9300
N3—H3N	0.8600	C11—H11	0.9300
N4—C11	1.330 (3)	C6A—H6A	0.9300
N4—C7	1.347 (3)	N3A—H3NA	0.8600
			
O1W—Mn1—N2	87.38 (6)	N1—C1—C2	123.8 (3)
O1W—Mn1—N4	85.96 (6)	N1—C1—H1	118.1
N2—Mn1—N4	69.62 (7)	C2—C1—H1	118.1
O1W—Mn1—N1	87.94 (5)	C3—C2—C1	118.4 (3)
N2—Mn1—N1	69.85 (7)	C3—C2—H2	120.8
N4—Mn1—N1	139.23 (7)	C1—C2—H2	120.8
O1W—Mn1—O2	83.70 (5)	C2—C3—C4	119.6 (2)
N2—Mn1—O2	152.55 (6)	C2—C3—H3	120.2
N4—Mn1—O2	135.11 (6)	C4—C3—H3	120.2
N1—Mn1—O2	83.90 (6)	C3—C4—C5	118.7 (3)
O1W—Mn1—O3	89.13 (6)	C3—C4—H4	120.6
N2—Mn1—O3	151.72 (6)	C5—C4—H4	120.6
N4—Mn1—O3	82.14 (6)	N1—C5—C4	122.1 (3)
N1—Mn1—O3	138.06 (6)	N1—C5—C6	117.1 (4)
O2—Mn1—O3	54.21 (5)	C4—C5—C6	120.8 (5)
O1W—Mn1—Cl1	179.07 (4)	N1—C5—N3A	116.5 (5)
N2—Mn1—Cl1	93.54 (4)	C4—C5—N3A	121.4 (5)
N4—Mn1—Cl1	94.25 (5)	N2—C6—C5	118.3 (7)
N1—Mn1—Cl1	92.48 (4)	N2—C6—H6	120.9
O2—Mn1—Cl1	95.52 (4)	C5—C6—H6	120.9
O3—Mn1—Cl1	90.00 (5)	C6A—C7—N4	118.6 (5)
Mn1—O1W—H1WA	124.5	C6A—C7—C8	119.5 (5)
Mn1—O1W—H1WB	116.1	N4—C7—C8	122.0 (3)
H1WA—O1W—H1WB	105.9	N4—C7—N3	115.6 (2)
N5—O2—Mn1	95.09 (10)	C8—C7—N3	122.4 (3)
N5—O3—Mn1	94.39 (10)	C9—C8—C7	119.1 (3)
C1—N1—C5	117.34 (19)	C9—C8—H8	120.4
C1—N1—Mn1	126.26 (14)	C7—C8—H8	120.4
C5—N1—Mn1	116.38 (16)	C8—C9—C10	119.5 (3)
N3—N2—C6	116.9 (6)	C8—C9—H9	120.2
N3A—N2—C6A	128.2 (6)	C10—C9—H9	120.2
N3—N2—Mn1	124.8 (4)	C9—C10—C11	119.0 (3)
N3A—N2—Mn1	120.1 (5)	C9—C10—H10	120.5
C6—N2—Mn1	118.4 (6)	C11—C10—H10	120.5
C6A—N2—Mn1	111.7 (5)	N4—C11—C10	122.7 (3)
N2—N3—C7	113.2 (4)	N4—C11—H11	118.6
N2—N3—H3N	123.4	C10—C11—H11	118.6
C7—N3—H3N	123.4	C7—C6A—N2	123.3 (8)
C11—N4—C7	117.6 (2)	C7—C6A—H6A	118.4
C11—N4—Mn1	126.03 (15)	N2—C6A—H6A	118.4
C7—N4—Mn1	116.15 (16)	N2—N3A—C5	117.1 (8)
O4—N5—O3	122.94 (16)	N2—N3A—H3NA	121.5
O4—N5—O2	120.77 (16)	C5—N3A—H3NA	121.5
O3—N5—O2	116.29 (15)		

### Hydrogen-bond geometry (Å, º)

**Table:**

*D*—H···*A*	*D*—H	H···*A*	*D*···*A*	*D*—H···*A*
O1*W*—H1*WA*···Cl1^i^	0.91	2.23	3.1225 (15)	170
O1*W*—H1*WB*···O2^ii^	0.87	1.92	2.7969 (19)	177
O1*W*—H1*WB*···N5^ii^	0.87	2.68	3.506 (2)	157
N3—H3*N*···Cl1^iii^	0.86	2.71	3.501 (7)	153
C1—H1···O2	0.93	2.53	3.140 (3)	124
C6—H6···Cl1^iii^	0.93	2.66	3.489 (11)	149
C8—H8···O4^iv^	0.93	2.54	3.290 (3)	138
C10—H10···Cl1^v^	0.93	2.83	3.669 (3)	152
C11—H11···O3	0.93	2.44	3.062 (3)	125

Symmetry codes: (i) *x*+1, *y*, *z*; (ii) −*x*+2, −*y*+1, −*z*+2; (iii) −*x*+1, −*y*+1, −*z*+1; (iv) *x*, *y*, *z*−1; (v) −*x*+1, −*y*, −*z*+1.
